# Unusual Course of Congenital Hypothyroidism and Route of the L-Thyroxine Treatment in a Preterm Newborn

**DOI:** 10.4274/jcrpe.1383

**Published:** 2014-09-05

**Authors:** Levent Korkmaz, Mustafa Ali Akın, Tamer Güneş, Ghaniya Daar, Osman Baştuğ, Ali Yıkılmaz, Selim Kurtoğlu

**Affiliations:** 1 Erciyes University Faculty of Medicine, Neonatology, Kayseri, Turkey; 2 Kayseri Training and Research Hospital, Neonatology Unit, Kayseri, Turkey; 3 Bozok University Faculty of Medicine, Department of Pediatrics, Yozgat, Turkey; 4 Erciyes University Faculty of Medicine, Department of Radiology, Kayseri, Turkey

**Keywords:** congenital hypothyroidism, L-thyroxine, prematurity

## Abstract

Congenital hypothyroidism (CH) is the most common endocrine pathology in neonates. Inappropriate treatment of CH is complicated by irreversible brain damage or low IQ score. Hormone replacement therapy with L-thyroxine (L-T4) is sufficient for a very large proportion of patients. However, during treatment, the patient needs to be carefully monitored for presence of factors which might affect the absorption or bio-availability of the drug as well as its dose. Herein, we report a preterm newborn with CH who presented with gastrointestinal problems mimicking necrotizing enterocolitis. The clinical course was also complicated by cholestasis. The L-T4 replacement treatment was switched from oral route to parenteral. After resolution of the cholestasis, L-T4 treatment was continued successfully by the oral route.

## INTRODUCTION

Congenital hypothyroidism (CH) is the most common endocrine disorder in neonates (1). Early detection and treatment as well as prevention from mental retardation have been made possible by mass screening programs. In general, patients with CH can be successfully treated with an appropriate L-thyroxine (L-T4) dose given daily per os (PO) (2). However, it is important that the L-T4 dose be carefully determined with consideration of any factor which might affect drug absorption and its bio-availability.

Herein, we present a newborn with CH who has required parenteral L-T4 treatment due to reduced absorption of orally given L-T4, caused by intrahepatic cholestasis.

## CASE REPORT

**Figure 1:**
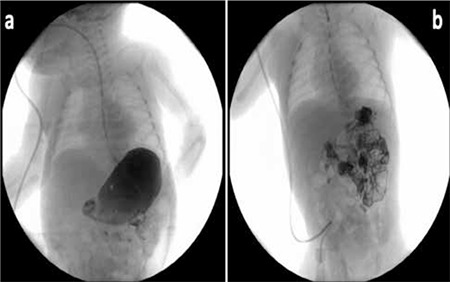
Upper gastrointestinal system radiography (UGIR) prior to L-thyroxine treatment: Upper GI series performed by using diluted barium via feeding catheter demonstrates a dilated and distended stomach at the 15th minute of the examination. There is slight opacification of the duodenum, and the emptying time of the stomach is markedly delayed. b) UGIR subsequent to L-thyroxine treatment: Follow-up GI series after medical treatment demonstrates that the emptying time of the stomach has improved. The stomach has almost totally emptied at the 15th minute of the examination with opacification of the duodenum and proximal jejunum

A preterm male newborn aged 33 gestational weeks born to a primiparous 24-year-old mother via C/S was admitted to our neonatology unit due to prematurity. His APGAR scores were 5 and 7 at the first and fifth minutes of birth, respectively. His weight was lower than the 10th percentile, and his head circumference and length were between the 50th and 75th percentiles. The mother’s medical history did not reveal presence of any chronic disease, drug, or X-ray exposure. There was no consanguinity between the parents. At admission, physical examination revealed normal findings except for a systolic murmur which was later identified to be due to a bicuspid aortic valve and patent foramen ovale by echocardiographic examination. When the baby stabilized, feeding was started via orogastric tube and changed to per oral feeding on the subsequent day. At the end of the first week, the infant was noted to develop abdominal distention and feeding intolerance with bilious residue. Gastric drainage was performed, and wide spectrum antibiotics and total parenteral nutrition (TPN) were started due to suspicion of necrotizing enterocolitis (NEC). At the second week of follow-up, plain X-ray of the abdomen was normal, abdominal distention decreased, and orogastric drainage was clear. However, the trials of re-feeding via orogastric tube with small volumes continued to be unsuccessful. Subsequently, significant gastro-esophageal reflux (GER), delayed and limited duodenal passage were detected by upper gastrointestinal system radiography (UGIR) (Figure 1). At this time, thyroid functions tests revealed a high serum thyroid-stimulating hormone (TSH) level (2331 mIU/L, normal range: 0.57-5.6 mIU/L), decreased free thyroxine level (fT4) (0.28 ng/dL, normal range: 0.88-1.72 ng/dL) and a decreased thyroglobulin level (<0.2 ng/mL, normal range: 0.73-84 ng/mL). Thyroid ultrasonography performed at this time revealed right-lobe hypoplasia and absence of the left lobe. 

L-T4 treatment (10 µg/kg/day PO) was started via orogastric tube. Within the five days of the NEC treatment, abdominal distention resolved and oral feedings were restarted. A control UGIR with contrast agent showed normal duodenal and intestinal passage although reflux and gastric distention continued. During follow-up, oral feedings did not reach sufficient volumes, therefore, TPN was continued. With oral L-T4 treatment, the fT4 levels reached normal levels and TSH levels decreased. At the end of the first month of the admission, hyperbilirubinemia, elevated transaminases (alanine transaminase and aspartate transaminase) and high cholestatic enzymes including gamma-glutamyl transferase and alkaline phosphatase (ALP) were detected. Serologic tests for viral hepatitis were negative except anti-CMV IgM. The lipid and protein contents of the TPN solution were reduced; ursodeoxycholic acid was started because of suspicion of cholestasis secondary to TPN solution infusion. PCR test could not be performed before the initiation of treatment. However, based on the clinical findings and serological test, ganciclovir treatment was started due to suspicion of cytomegalovirus hepatitis. Following the development of this hepatitis episode, thyroid function tests were found to be altered, with an increase in TSH levels and a decrease in fT4 levels. Hence, the dose of the L-T4 was increased to 15 µg/kg/day, and subsequently to 20 µg/kg/day. However, fT4 and TSH levels failed to reach normal ranges. Intravenous (IV) L-T4 (10 µg/kg/day) replacement was started. After a week of L-T4 treatment, an increase in serum fT4 level and a significant decrease in serum TSH level were noted. Additionally, improvement of the patient’s impaired liver enzyme levels and decrease in his direct bilirubin level were observed. Oral feedings were started and well tolerated. When the daily feeding volumes reached adequate levels, TPN replacement was stopped (on the 40th day of admission). The L-T4 treatment was also changed to PO treatment (15 µg/kg/day). A week after this treatment, the CMV-polymerase chain reaction was negative (<235 genome copies/mL). The ganciclovir therapy was continued for 21 days. Thyroid hormone levels and treatment modalities during hospitalization are summarized in (Table 1).

When the patient recovered from cholestasis, the oral L-T4 dose was reduced to 5 µg/kg/day and the infant was discharged. During the 18 months of follow-up, no increase in oral L-T4 dose has been required.

## DISCUSSION

This case report showed that CH can rarely present with severe GER and delayed and limited UGIS motility. Additionally, some clinical situations such as cholestasis, which may affect the bio-availability of the drug during PO L-T4 replacement therapy, may also develop and L-T4 replacement therapy may be essential for achieving euthyroidic state.

Gastrointestinal absorption is one of the important factors determining the success of PO L-T4 therapy. Gastric acid secretion plays a very important role in the intestinal absorption of L-T4. According to earlier studies, the stomach, duodenum, upper and lower jejuno-ileum are the sites which are mainly responsible for T4 absorption. T4 absorption takes place in all segments of the small intestines equally but decreases in the colon (3,4). Therefore, some gastrointestinal diseases including atrophic gastritis, Helicobacter pylori infections, cystic fibrosis, celiac disease, inflammatory bowel diseases, lactose intolerance, short bowel syndrome, cirrhosis, and giardiasis can cause failure of PO L-T4 therapy by impairing its absorption (4). Additionally, a great number of drugs such as cholestiramine, anti-acid substances including aluminium hydroxide, proton-pump inhibitors, sucralphate, ferrous sulphate, calcium carbonate, statins, orlistate, ion-changing resins, soy bean, fiber-rich foods, overconsumption of acidic fruit juice, and simethicone may interact the with L-T4 or decrease its absorption (3,5). Moreover, the gastrointestinal signs or symptoms of CH may sometimes be missed or misdiagnosed in the neonatal period. Feeding difficulties and decreased peristalsis which can cause mechanical ileus can be improved with L-T4 replacement treatment. Our patient was not given any medications which affect the gastointestinal absorption of L-T4. Despite resolution of suspected NEC, the patient’s oral enteral feedings did not reach an optimal volume. We attributed the failure to reach optimal oral feeding volumes to disturbed GIS functions resulting from CH. Additionally, either weak meal-stimulated gastric acid secretory response or non-optimal feeding volume as well as incapability of hydrogen ion secretion in preterms might affect the gastric acid secretion causing malabsorbtion of L-T4. 

The relationship between cholestasis and hypothyroidism is well defined. Deficiency of thyroid hormones may cause a lowering of the bile flow and vice versa (6,7). In our patient, the reasons of the cholestasis could be prematurity, ineffective L-T4 treatment, prolonged TPN, the simultaneously detected CMV hepatitis, or the collective effect of all these factors. The cholestasis might have caused or worsened the absorption of orally administered L-T4 or its utilization. We therefore had to switch from the oral route to parenteral L-T4 treatment. 

In conclusion, the gastrointestinal symptoms in CH may at times lead to perplexing symptoms in neonates, especially in preterm newborns. During the hospital stay of preterm newborns diagnosed with CH and treated with PO L-T4, close monitoring of the thyroid functions is essential due to the vulnerability of the hypothalamo-pituitary-thyroid axis and to possibility of development of clinical conditions which affect the absorption and bio-availability of orally administered L-T4.

## Figures and Tables

**Table 1:**
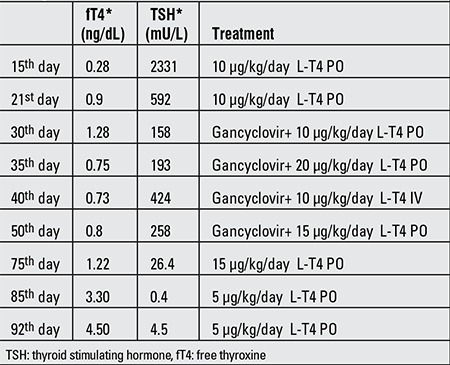
TSH and fT4 levels of the patient and treatment modalities during hospitalization
